# Peek Inside the Water Mixtures of Ionic Liquids at Molecular Level: Microscopic Properties Probed by EPR Spectroscopy

**DOI:** 10.3390/ijms222111900

**Published:** 2021-11-02

**Authors:** Mikhail Yu. Ivanov, Yuliya F. Polienko, Igor A. Kirilyuk, Sergey A. Prikhod’ko, Nicolay Yu. Adonin, Matvey V. Fedin

**Affiliations:** 1 International Tomography Center SB RAS, Institutskaya Street 3a, 630090 Novosibirsk, Russia; 2 N. N. Vorozhtsov Novosibirsk Institute of Organic Chemistry SB RAS, Lavrentiev Avenue 9, 630090 Novosibirsk, Russia; polienko@nioch.nsc.ru (Y.F.P.); kirilyuk@nioch.nsc.ru (I.A.K.); 3 Boreskov Institute of Catalysis SB RAS, Lavrentiev Avenue 5, 630090 Novosibirsk, Russia; spri@catalysis.ru (S.A.P.); adonin@catalysis.ru (N.Y.A.); 4 Novosibirsk State University, Pirogova Street 2, 630090 Novosibirsk, Russia

**Keywords:** ionic liquids, EPR spectroscopy, nanostructure, emulsions, green chemistry

## Abstract

Many ionic liquids (ILs) can be mixed with water, forming either true solutions or emulsions. This favors their applications in many respects, but at the same time might strongly alter their physicochemical properties. A number of methods exist for studying the macroscopic properties of such mixtures, whereas understanding their characteristics at micro/nanoscale is rather challenging. In this work we investigate microscopic properties, such as viscosity and local structuring, in binary water mixtures of IL [Bmim]BF_4_ in liquid and glassy states. For this sake, we use continuous wave and pulse electron paramagnetic resonance (EPR) spectroscopy with dedicated spin probes, located preferably in IL-rich domains or distributed in IL- and water-rich domains. We demonstrate that the glassy-state nanostructuring of IL-rich domains is very similar to that in neat ILs. At the same time, in liquid state the residual water makes local viscosity in IL-rich domains noticeably different compared to neat ILs, even though the overwhelming amount of water is contained in water-rich domains. These results have to be taken into account in various applications of IL-water mixtures, especially in those cases demanding the combinations of optimum micro- and macroscopic characteristics.

## 1. Introduction

Ionic Liquids (ILs) exhibit a number of unusual and advanced properties making them perspective media for many chemical processes in various fields of modern science and technology [1,2,3]. ILs are suitable for such applications as electrolytes in batteries, catalysis, solvents in liquid–liquid extractions; they have also attracted considerable attention as green chemistry reaction media for many organic reactions [4,5,6,7,8,9,10]. The most unusual characteristic of ILs is their nanoscale self-organization, leading to a formation of various heterogeneities [11,12].

The presence of water strongly affects the physical and chemical properties of ILs, such as reactivity, electric conductivity, polarity, self-organization, and formation of heterogeneities [13,14,15,16,17]. For instance, obtaining reproducible results on neat ILs requires eliminating of traces of water down to ~200 ppm [18,19]. At the same time, IL/water mixtures find their own decent applications, especially in biologically-relevant systems [20,21,22,23]. In many cases ILs in water form structures on nano- to micrometer scale somewhat resembling micelles [24,25]. In some other cases, IL-rich and water-rich domains are formed, leading to certain dynamic heterogeneities in liquid state [26,27].

Particularly interesting results on heterogeneous structures in IL/water mixtures were recently obtained using electron microscopy at room temperature [28]. Different micrometer and submicrometer scale structures were observed in mixtures of [Bmim]BF_4_ with water depending on the ratio of components. Bubbles of water in IL bulk, channels and various meshworks could be clearly identified. These recent observations brought us to an interesting and potentially important question—what are the physical properties inside such well-defined heterogeneities? The domains with the size larger than 100 nm contain many molecules of one kind (water or IL), and might resemble to certain extent the corresponding bulk solvent. Thus, are the physical parameters sensed by small molecules inside such IL or water nanodomains drastically different from parameters of individual neat components?

Most of physical methods such as viscosimetry, dielectric spectroscopy, X-ray and neutron scattering, and Raman spectroscopy measure bulk properties of such mixtures as IL/water. However, if reactants are preferably localized in IL (or water) domains, the reaction pathways and products can be significantly altered. Moreover, in some cases it would be much easier to use IL mixtures with water instead of neat ILs, if this would not sacrifice any target products or results. For instance, this is the case for flow reactors, where high-viscosity ILs would exhibit strong hydrodynamic resistance, whereas for IL/water mixtures it will be drastically reduced. In addition, the necessity to carry out chemical syntheses in totally anhydrous ILs is energy-demanding on its own.

Taking into account all above, in this work we have designed and performed a case study of microscopic physicochemical properties in water mixtures of prototypical IL [Bmim]BF_4_. For this sake, we used the advanced electron paramagnetic resonance (EPR) spectroscopy and two dedicated spin probes, one of which had preferred localization in IL domains, whereas another one localized in both IL and water. Applications of EPR to study ILs grew up during past two decades [19,29,30,31,32,33,34,35,36], and even the mixtures [Bmim]BF_4_/water were addressed in a few works [30,31,37]. However, recent experimental results of Ref. [28] strongly encouraged the idea that domain-specific probes can provide enhanced and unique information on the inner properties of each domain. Moreover, in a recent series of works we developed a complex and highly informative EPR approach to characterize heterogeneities in ILs [37,38,39,40,41,42,43,44]. In this work we for the first time exploit this methodology along with dedicated nitroxide probes, and apply it to [Bmim]BF_4_/water mixtures, since exactly these mixtures were recently studied in Ref. [28]. Such approach allowed us to peek inside the IL-rich micro/nanodomains within IL/water mixtures, as is described in detail in the following sections.

## 2. Experimental

Ionic liquid 1-butyl-3-methylimidazolium tetrafluoroborate ([Bmim]BF_4_) was synthesized according to the procedure described in Ref. [45] in Boreskov Institute of Catalysis SB RAS (see Supplementary Materials).

Spin probes—nitroxide radicals K1 [46] and K2 [47]—were prepared according to the literature protocols in the Novosibirsk Institute of Organic Chemistry SB RAS (see Supplementary Materials for details). Both radicals are well soluble in [Bmim]BF_4_; however, the solubility of K2 in water is a few orders of magnitude lower compared to K1. Therefore, K1 was targeted as spin probe not specific for the components of the studied IL/water mixture, whereas K2 was selected as spin probe with preferred localization in IL domains. ESEEM experiments done in this work have fully confirmed these expectations (see below).

Pulse EPR measurements were performed using a commercial Bruker Elexsys E580 spectrometer at X-band. The spectrometer was equipped with an Oxford Instruments temperature control system (4−300 K). The echo-detected EPR spectra and phase memory times were recorded using the standard two-pulse echo sequence with pulse lengths being typically 100 ns for π and 50 ns for π/2 pulses. Three-pulse Electron Spin Echo Envelope Modulation (ESEEM) experiments used pulse sequence [π/2 – τ – π/2 – T – π/2 – echo], with 10 ns pulses, τ = 180 ns, T = 600 ns, and temperature 20 K. Continuous wave (CW) EPR measurements were done using an X-band Bruker EMX spectrometer (9 GHz). All spectral simulations were done using EasySpin [48].

## 3. Results and Discussion

We used a combination of CW and pulse EPR in this study. CW EPR allowed us to investigate IL-water mixtures at room temperature, i.e., in practical conditions for potential applications, and study local viscosity in IL-rich domains of mixtures compared to that of neat IL. Pulse EPR was used for two purposes: (i) to probe local environment of the radical, whether it contains water or not, and (ii) to assess nanostructural properties around spin probe and compare them with those of neat bulk IL.

**Scheme 1:**
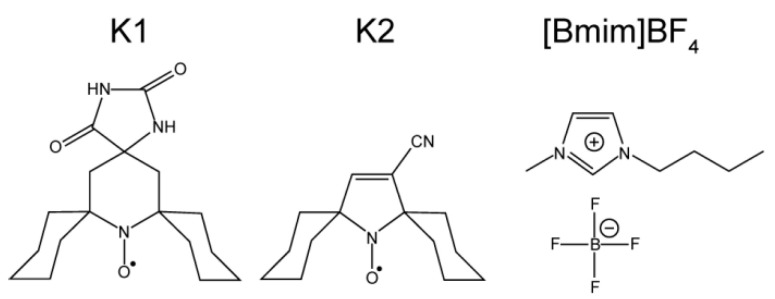
Structures of spin probes K1 and K2, and ionic liquid [Bmim]BF_4_.

As was already mentioned above, recent studies of [Bmim]BF_4_/water mixtures by electron microscopy have revealed a clearly defined micrometer-scale domains of water in IL for the water content up to ~20 vol.% [28]. We selected two dedicated spin probes, denoted as K1 and K2, with the purpose that K2 should strongly prefer hydrophobic (IL-rich) domains, whereas K1 should locate in hydrophilic water domains as well (Scheme 1). Simultaneously, both probes have suitable spectroscopic (relaxation) properties due to the absence of methyl groups adjacent to NO moiety [46,47,49].

First of all, we verified whether or not this preferred localization in one type of domains does occur for K2. For this sake, we used pulse EPR spectroscopy ESEEM (Electron Spin Echo Envelope Modulation) on shock-frozen samples. ESEEM allows detection of nuclear spins in the radius of ~1 nm around spin probe [50]. Most abundant nuclei around probe are, of course, protons; in order to distinguish protons of water from protons of IL, we used deuterated water (D_2_O) in these experiments [50,51].

**Figure 1:**
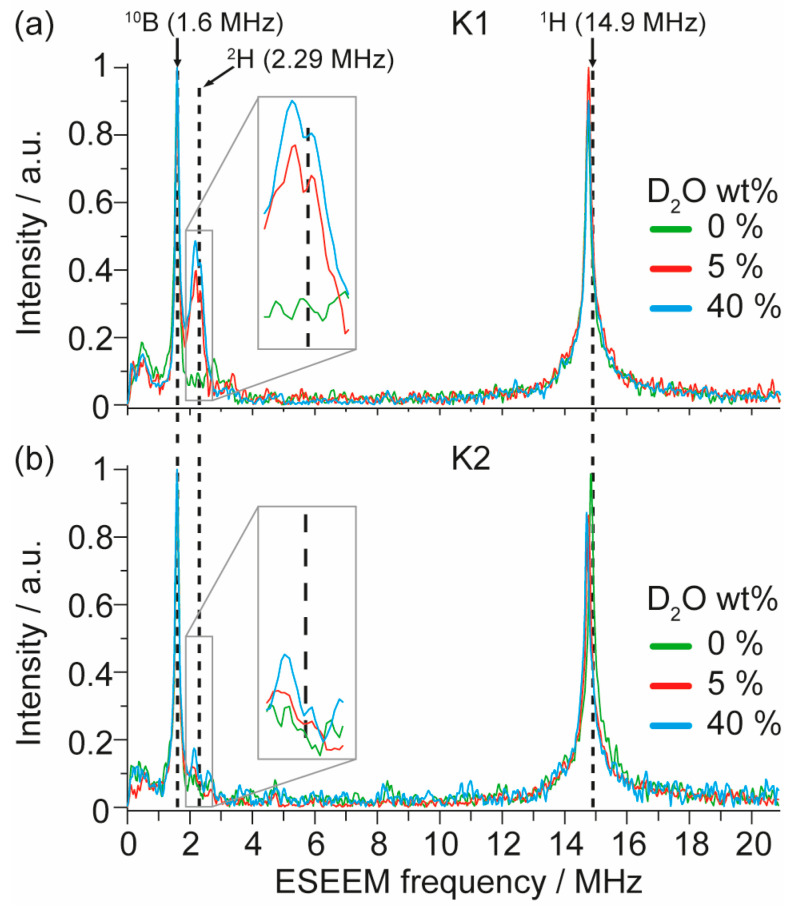
Three-pulse ESEEM spectra detected for K1 (**a**) and K2 (**b**) probes dissolved in various D_2_O/[Bmim]BF_4_ mixtures (indicated in legend). All spectra are normalized to ^10^B signal intensity.

Figure 1a shows an appearance of deuterium peak (2.29 MHz for distant deuterons, for the X-band EPR) for K1 probe dissolved in IL/water mixtures with 5 and 40% (w/w) of D_2_O, while this peak is absent in neat IL containing no deuterium atoms. At the same time, when K2 is dissolved in similar D_2_O/[Bmim]BF_4_ mixtures no deuterium peak is found neither for 5%, nor for 40% of water (Figure 1b). This clearly means that K2 is preferably located in water-free compartments of IL, i.e., in IL domains. This also confirms that such water-free nanodomains of apparently pure IL do exist in the IL/water mixtures. At the same time, K1 is well accessible for the D_2_O molecules, meaning that it locates either solely in water compartments, or, more realistically, is distributed between both water and IL domains. Finally, Figure S1 (Supplementary Materials) justifies the assignments of all other peaks in ESEEM spectrum.

Previously, we developed a potent complex EPR approach to characterize local nanostructuring of solvent molecules around spin probe [40,41,42,43,44]. In particular, pulse EPR was used to monitor (transverse) spin relaxation induced by so-called stochastic molecular librations of the surrounding matrix (see Supplementary Materials for details) [52]. Such librations (small-angle wobbling) of matrix are translated onto the radical probe and cause its own librational motion [53,54]. As a result, transverse relaxation time *T*_2_ significantly depends on the spectral position where it is recorded (denoted as I and II) and is strongly interconnected with libration amplitude (α) and correlation time (*τ*_c_) of spin probe. The experimentally obtained function *L*(*T*)*∝*(1/*T*_2_^(*II*)—^1/*T*_2_^(*I*)^) in a simplified way, can be interpreted as a characteristic of local density or rigidity of the medium, allowing spin probe to wobble with larger or smaller amplitude (see Supplementary Materials for details).

**Figure 2:**
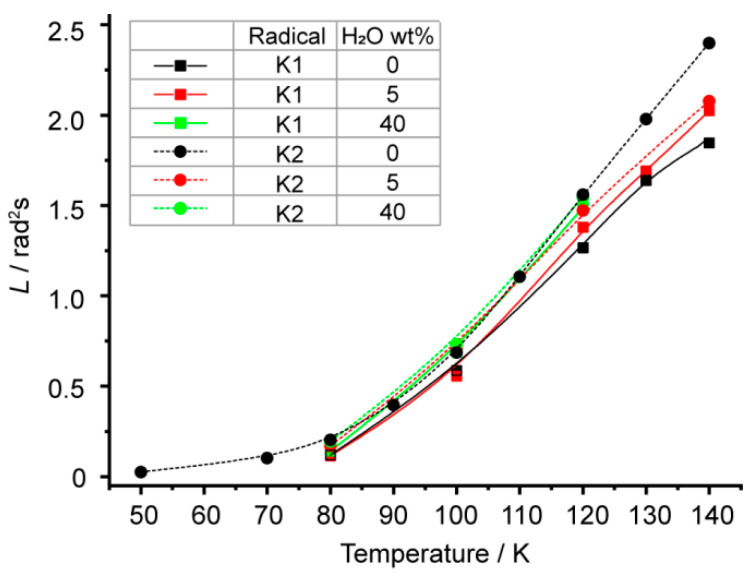
Temperature dependence of the motional parameter *L* for used nitroxide spin probe in ILs and IL/water mixtures. Color lines are guides for the eye.

Figure 2 compares *L*(*T*) curves obtained for neat IL and IL/water mixtures using K1 and K2 probes. It is important to note that librations (i.e., nonzero *L*(*T*)) can be observed only in glassy state, not in crystalline state; therefore, for both radicals K1 and K2 we observe only those radicals located in IL-rich domains. According to ESEEM data above, in case of K2 this is nearly all radicals, whereas for K1 this corresponds only to a fraction of radicals.

It is clear that all *L*(*T*) dependences in Figure 2 are similar, meaning that spin probe located in the IL-rich domains of IL/water mixtures senses closely the same nanostructuring as in the neat IL. As we have demonstrated previously [39], the local surrounding of spin probes in ILs is very loose compared to normal glasses such as water/glycerol or sucrose octaacetyl acetate. Thus, this loose micelle-like nanoenvironment in IL-rich nanodomains is not essentially disturbed by the presence of water in binary mixture. The results shown in Figure 2 agree well with previously obtained results using another spin probe in Ref. [37].

**Figure 3:**
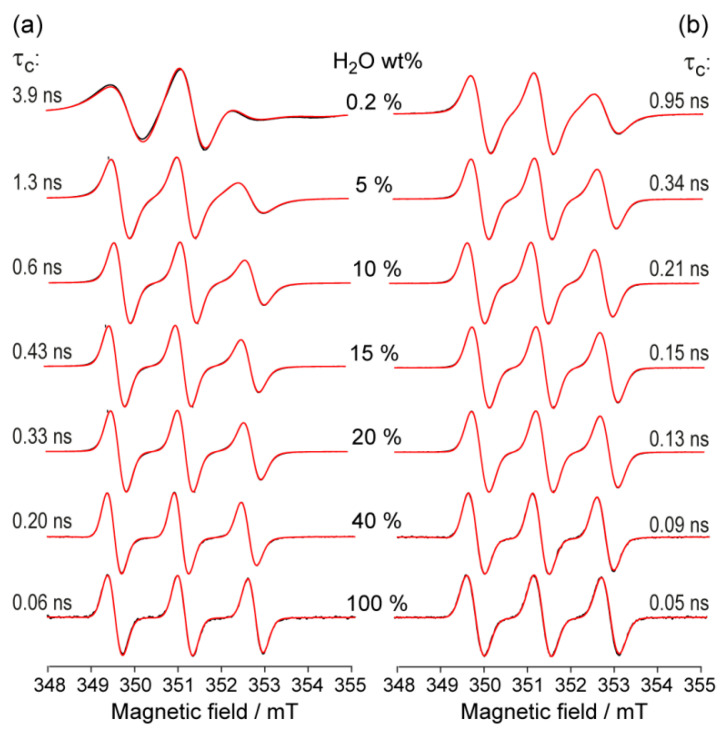
CW EPR spectra of K1 (**a**) and K2 (**b**) radicals dissolved in H_2_O/[Bmim]BF_4_ mixtures with water concentration depicted (in wt%) at room temperature. Black curves show experimental data, red curves–simulation results. The values of τ_c_ are shown for each spectrum. Other simulation parameters are given in Supplementary Materials.

Finally, we performed room-temperature CW EPR studies of IL/water mixtures in liquid state, in order to compare local viscosity sensed by domain-specific spin probes in neat IL vs. IL/water mixtures (Figure 3). Since ESEEM indicates strong preference for K2 to localize in IL-rich domains, it would be reasonable to expect that the EPR spectrum remains essentially the same at water contents 0–40%. In case of K1, which efficiently occupies water-rich domains as well, spectral changes might be significant. This is because the shape of the X-band EPR spectrum of nitroxide is extremely sensitive to the speed of rotation (rotational correlation time, τ_c_), which, in turn, depends on the viscosity of the medium. Since the viscosity of IL is much higher than that of water, drastically different spectra are anticipated in IL and in water.

**Figure 4:**
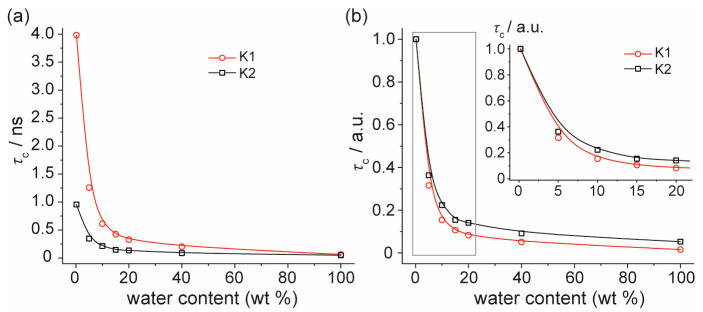
(**a**) Rotational correlation time τ_c_ of K1 and K2 radicals dissolved in [Bmim]BF_4_/water mixtures vs. water content. Values are obtained from simulations of CW EPR spectra shown in Figure 3. (**b**) Values of τ_c_ normalized to 1 at zero water content. Color lines are guides for the eye.

Indeed, in case of K1 the observations correspond to the expectation (Figure 3a). The spectra in pure IL and water are drastically different, and τ_c_ obtained from simulations differs by more than an order of magnitude (see τ_c_ values in Figure 3 and the full list of simulation parameters in Supplementary Materials). In case of IL-water mixtures, intermediate spectral shapes are observed. In all cases, however, the spectrum is well simulated by a single component; accounting of two components of different viscosity, e.g., that of IL and water, is not justified and provides poorer agreement with experiment. Therefore, either there is a distribution of local environments and simulation yields some mean τ_c_ and viscosity, or fast diffusion of radicals between water-rich and IL-rich domains takes place, so that the average values are observed. As the weight of low-viscosity water is increasing, the mean τ_c_ decreases (see Figure 4).

However, surprisingly, the dependence observed for K2 is essentially similar to that found for K1 (Figure 3b and Figure 4a). Note that the size of the K2 radical is much smaller than that of K1. The rotational correlation time τ_c_ can be estimated using the Stokes-Einstein-Debye expression τ_c_ = (4π*r*^3^η)/(3*kT*), where *r* is the radius of the particle, η is the viscosity, *k*–Boltzman constant, and *T*–temperature. Therefore, ~6 times larger τ_c_ value for K1 in neat IL compared to K2 is reasonable and corresponds to ~6^1/3^ ≈ 1.8 times difference in the hydrodynamic radii. However, the normalized dependence of τ_c_ on water content is very similar for K1 and K2 (see Figure 4b). Since τ_c_ is directly proportional to viscosity η, normalization eliminates the dependence on radical radius (*r*^3^); therefore, the data of Figure 4b indicate that both radicals K1 and K2 exhibit closely similar local viscosity in the whole range of water contents. Taking into account the results of ESEEM experiments (Figure 1) showing the absence of water in the vicinity of K2 and its presence around K1, closely similar behaviors in Figure 4b need explanation.

In fact, the dependence of τ_c_ on water content decays slightly faster for K1 compared to K2 (Figure 4b), showing that the presence of added water is sensed by K2 to a lesser extent compared to K1, as is expected. However, still the observed dependence for K2 is dramatic; if K2 would mainly reside in water-free domains with a small contribution of its presence in water-rich domains, one would expect zero to very weak dependence of τ_c_ on water content. As was already mentioned above, the separation of water and [Bmim]BF_4_ at microscopic scale was recently evidenced by clear FE-SEM images at 0–20 wt.% of water [28]. At ~5% the clear droplets of water with dimensions of ~1 μm were observed in the bulk of IL, and at 20% the complicated meshworks of comparable size were formed. These conditions must be fulfilled in our experiments as well, and microscopic-scale separation of water and IL are to be expected; yet CW EPR data of Figure 3 and Figure 4 do not indicate any separation.

The apparent contradiction between CW EPR (Figure 3 and Figure 4) and ESEEM (Figure 1) in our paper, as well as apparent contradiction of our CW EPR data with SEM images of Ref. [28], can be explained as follows. It is well known that tiny amounts of water in ILs can drastically modify their physicochemical properties, and ‘neat IL properties’ assume residual water contents less than ~200 pm [18,19]. If IL-rich domains in binary mixtures still contain some residual water in certain amounts, the viscosity can be very different compared to the neat IL case, and this should be sensed by CW EPR. However, it is unlikely that small amounts of residual water are visible in SEM images of IL-rich regions presented in Ref. [28]. Similarly, ESEEM would not sense amounts of D_2_O less than a few percent around radical, as is evident from Figure 1a at given signal-to-noise ratio. In fact, small D_2_O peak is even visible for K2 at 40% of water (Figure 1b), but this should not be overinterpreted and might be alternatively assigned to a small fraction of K2 localized in water domains.

Finally, it was interesting to verify whether this interpretation agrees with the SEM images of Ref. [28] or not. For this sake we have estimated the ratio of areas corresponding to water droplets and IL bulk at ~5% of water content (Figure 1c of ref. [28]), because this image has most clearly defined boundaries between water and IL. The analysis of areas (see Supplementary Materials) shows that well-defined water droplets contain only ~3% of overall area, and the remaining ~2% can potentially be semi-homogeneously mixed with remaining 95% of IL. In this case, the content of water in IL-rich domains should be around 2%. Although such estimation is very crude, there is general agreement between our rationale and data of Ref. [28].

Thus, we suggest that the whole dataset should be understood as follows. The binary mixtures of [Bmim]BF_4_ with water exhibit strongly heterogeneous structure on microscopic scale (~1–10 μm), where water-rich and IL-rich domains are clearly present with well-defined boundaries [28]. However, the separation of mixture components is not complete, and residuals of water are also present in IL-rich domains, strongly modifying the microscopic properties sensed by molecules dissolved in such domains. Such information on physicochemical properties of micrometer-sized domains has become available here due to the application of dedicated domain-specific spin probes and complex of advanced CW/pulse EPR techniques.

## 4. Conclusions

Small contents of water can significantly influence physicochemical properties of ionic liquids, and elimination of water residuals is an energy-consuming process. At the same time, neat ILs can face other kinds of difficulties in applications as green solvents, e.g., owing to their very high viscosity. Therefore, detailed understanding of local nanoscale environments around solutes in binary IL/water mixtures is of great importance, as it allows prediction whether water residuals are crucial or not for particular applications.

In this work we applied pulse and continuous wave EPR to various binary mixtures of prototypical IL [Bmim]BF_4_ with water, which have been previously demonstrated to form water droplets and meshworks in the bulk of IL [28]. Using two spin probes that are preferably localized in IL or in water domains we have shown that IL-rich micrometer-sized domains in these mixtures are indeed composed predominantly by IL molecules and display similar nanostructure. However, residual water is still ‘homogeneously’ present in such IL-rich domains in amounts sufficient to drastically reduce their viscosity. This information is obtained at the nanometer scale and is directly relevant to the viscosity sensed by small solute molecules during reactions in IL/water mixtures.

We have selected [Bmim]BF_4_ for this study, because this IL is miscible with water at any ratios; however, the impact of water residuals in other ILs on the properties of IL-rich domains can also be investigated using the same approach in the future. Therefore, we believe that the insights of this work will aid in adjusting optimum conditions for the practical use of IL/water mixtures as green solvents and for their other prospective applications.

## Supplementary Materials

The following are available online at https://www.mdpi.com/article/10.3390/ijms222111900/s1, Synthesis of ionic liquid [Bmim]BF_4_. Synthesis of the spin probes K1 and K2. Auxiliary ESEEM data. Continuous wave EPR spectra simulation. EPR of stochastic molecular librations in a nutshell. FE-SEM images analyses [28,45,46,47,48,54,55,56].

## References

[1] Welton T. (1999). Room-Temperature Ionic Liquids. Solvents for Synthesis and Catalysis. Chem. Rev..

[2] Hallett J.P., Welton T. (2011). Room-Temperature Ionic Liquids: Solvents for Synthesis and Catalysis. 2. Chem. Rev..

[3] Weingärtner H. (2008). Understanding Ionic Liquids at the Molecular Level: Facts, Problems, and Controversies. Angew. Chem. Int. Ed..

[4] Wasserscheid P., Keim W. (2000). Ionic Liquids - New “Solutions” for Transition Metal Catalysis. Angew. Chemie Int. Ed..

[5] Zhao D., Wu M., Kou Y., Min E. (2002). Ionic liquids: Applications in catalysis. Catal. Today.

[6] Dai C., Zhang J., Huang C., Lei Z. (2017). Ionic Liquids in Selective Oxidation: Catalysts and Solvents. Chem. Rev..

[7] Watanabe M., Thomas M., Zhang S., Ueno K., Yasuda T., Dokko K. (2017). Application of Ionic Liquids to Energy Storage and Conversion Materials and Devices. Chem. Rev..

[8] Shang D., Liu X., Bai L., Zeng S., Xu Q., Gao H., Zhang X. (2017). Ionic liquids in gas separation processing. Curr. Opin. Green Sustain. Chem..

[9] Zhang M., Ettelaie R., Yan T., Zhang S., Cheng F., Binks B.P., Yang H. (2017). Ionic Liquid Droplet Microreactor for Catalysis Reactions Not at Equilibrium. J. Am. Chem. Soc..

[10] Egorova K.S., Gordeev E.G., Ananikov V.P. (2017). Biological Activity of Ionic Liquids and Their Application in Pharmaceutics and Medicine. Chem. Rev..

[11] Wang Y.-L., Li B., Sarman S., Mocci F., Lu Z.-Y., Yuan J., Laaksonen A., Fayer M.D. (2020). Microstructural and Dynamical Heterogeneities in Ionic Liquids. Chem. Rev..

[12] Hayes R., Warr G.G., Atkin R. (2015). Structure and Nanostructure in Ionic Liquids. Chem. Rev..

[13] Wang J., Tian Y., Zhao Y., Zhuo K. (2003). A volumetric and viscosity study for the mixtures of 1-n-butyl-3-methylimidazolium tetrafluoroborate ionic liquid with acetonitrile, dichloromethane, 2-butanone and N, N ? dimethylformamide. Green Chem..

[14] Widegren J., Saurer E.M., Marsh K.N., Magee J.W. (2005). Electrolytic conductivity of four imidazolium-based room-temperature ionic liquids and the effect of a water impurity. J. Chem. Thermodyn..

[15] Najdanovic-Visak V., Esperança J.M.S.S., Rebelo L.P.N., da Ponte M.N., Guedes H.J.R., Seddon K.R., de Sousa H.C., Szydlowski J. (2003). Pressure, Isotope, and Water Co-solvent Effects in Liquid−Liquid Equilibria of (Ionic Liquid + Alcohol) Systems. J. Phys. Chem. B.

[16] Rebelo L.P.N., Najdanovic-Visak V., Visak Z.P., da Ponte M.N., Szydlowski J., Cerdeiriña C.A., Troncoso J., Romaní L., Esperança J.M.S.S., Guedes H.J.R. (2004). A detailed thermodynamic analysis of [C4mim][BF4] + water as a case study to model ionic liquid aqueous solutions. Green Chem..

[17] Zhang L., Xu Z., Wang Y., Li H. (2008). Prediction of the Solvation and Structural Properties of Ionic Liquids in Water by Two-Dimensional Correlation Spectroscopy. J. Phys. Chem. B.

[18] Strehmel V., Rexhausen H., Strauch P., Strehmel B. (2010). Temperature Dependence of Interactions Between Stable Piperidine-1-yloxyl Derivatives and a Semicrystalline Ionic Liquid. ChemPhysChem.

[19] Kattnig D.R., Akdoğan Y., Lieberwirth I., Hinderberger D. (2013). Spin probing of supramolecular structures in 1-butyl-3-methyl-imidazolium tetrafluoroborate/water mixtures. Mol. Phys..

[20] Frade R.F., Rosatella A.A., Marques C.S., Branco L.C., Kulkarni P.S., Mateus N.M.M., Afonso C.A.M., Duarte C.M. (2009). Toxicological evaluation on human colon carcinoma cell line (CaCo-2) of ionic liquids based on imidazolium, guanidinium, ammonium, phosphonium, pyridinium and pyrrolidinium cations. Green Chem..

[21] Viciosa M.T., Santos G., Costa A., Danède F., Branco L.C., Jordão N., Correia N.T., Dionísio M. (2015). Dipolar motions and ionic conduction in an ibuprofen derived ionic liquid. Phys. Chem. Chem. Phys..

[22] Takekiyo T., Yamazaki K., Yamaguchi E., Abe H., Yoshimura Y. (2012). High Ionic Liquid Concentration-Induced Structural Change of Protein in Aqueous Solution: A Case Study of Lysozyme. J. Phys. Chem. B.

[23] Takekiyo T., Koyama Y., Yamazaki K., Abe H., Yoshimura Y. (2013). Ionic Liquid-Induced Formation of the α-Helical Structure of β-Lactoglobulin. J. Phys. Chem. B.

[24] Gutowski K.E., Broker G.A., Willauer H.D., Huddleston J.G., Swatloski R.P., Holbrey J., Rogers R.D. (2003). Controlling the Aqueous Miscibility of Ionic Liquids: Aqueous Biphasic Systems of Water-Miscible Ionic Liquids and Water-Structuring Salts for Recycle, Metathesis, and Separations. J. Am. Chem. Soc..

[25] Singh T., Kumar A. (2007). Aggregation Behavior of Ionic Liquids in Aqueous Solutions: Effect of Alkyl Chain Length, Cations, and Anions. J. Phys. Chem. B.

[26] Sturlaugson A.L., Fruchey K.S., Fayer M.D. (2012). Orientational Dynamics of Room Temperature Ionic Liquid/Water Mixtures: Water-Induced Structure. J. Phys. Chem. B.

[27] Schröder C., Hunger J., Stoppa A., Buchner R., Steinhauser O. (2008). On the collective network of ionic liquid/water mixtures. II. Decomposition and interpretation of dielectric spectra. J. Chem. Phys..

[28] Kashin A., Galkin K.I., Khokhlova E.A., Ananikov V.P. (2016). Direct Observation of Self-Organized Water-Containing Structures in the Liquid Phase and Their Influence on 5-(Hydroxymethyl)furfural Formation in Ionic Liquids. Angew. Chem. Int. Ed..

[29] Akdoğan Y., Heller J., Zimmermann H., Hinderberger D. (2010). The solvation of nitroxide radicals in ionic liquids studied by high-field EPR spectroscopy. Phys. Chem. Chem. Phys..

[30] Kattnig D.R., Hinderberger D. (2012). Temperature-Dependent Formation and Transformation of Mesostructures in Water-Ionic Liquid Mixtures. Chem. – Asian J..

[31] Kattnig D.R., Akdoğan Y., Bauer C., Hinderberger D. (2012). High-Field EPR Spectroscopic Characterization of Spin Probes in Aqueous Ionic Liquid Mixtures. Z. Phys. Chem..

[32] Mladenova B.Y., Chumakova N.A., Pergushov V.I., Kokorin A., Grampp G., Kattnig D.R. (2012). Rotational and Translational Diffusion of Spin Probes in Room-Temperature Ionic Liquids. J. Phys. Chem. B.

[33] Mladenova B.Y., Kattnig D.R., Grampp G. (2011). Room-Temperature Ionic Liquids Discerned Via Nitroxyl Spin Probe Dynamics. J. Phys. Chem. B.

[34] Strehmel V., Laschewsky A., Stoesser R., Zehl A., Herrmann W. (2006). Mobility of spin probes in ionic liquids. J. Phys. Org. Chem..

[35] Stoesser R., Herrmann W., Zehl A., Strehmel V., Laschewsky A. (2006). ESR Spin Probes in Ionic Liquids. ChemPhysChem.

[36] Strehmel V. (2012). Radicals in Ionic Liquids. ChemPhysChem.

[37] Ivanov M., Prikhod’Ko S.A., Adonin N.Y., Fedin M.V. (2019). Structural Anomalies in Binary Mixtures of Ionic Liquid [Bmim]BF4 with Water Studied by EPR. J. Phys. Chem. B.

[38] Ivanov M., Veber S., Prikhod’Ko S.A., Adonin N.Y., Bagryanskaya E., Fedin M. (2015). Probing Microenvironment in Ionic Liquids by Time-Resolved EPR of Photoexcited Triplets. J. Phys. Chem. B.

[39] Ivanov M., Prikhod’Ko S.A., Adonin N.Y., Bagryanskaya E., Fedin M.V. (2017). Influence of C2-Methylation of Imidazolium Based Ionic Liquids on Photoinduced Spin Dynamics of the Dissolved ZnTPP Studied by Time-Resolved EPR. Z. Phys. Chem..

[40] Ivanov M.Y., Krumkacheva O.A., Dzuba S.A., Fedin M.V. (2017). Microscopic rigidity and heterogeneity of ionic liquids probed by stochastic molecular librations of the dissolved nitroxides. Phys. Chem. Chem. Phys..

[41] Ivanov M.Y., Prikhod’Ko S.A., Adonin N.Y., Kirilyuk I.A., Adichtchev S.V., Surovtsev N.V., Dzuba S.A., Fedin M.V. (2018). Structural Anomalies in Ionic Liquids near the Glass Transition Revealed by Pulse EPR. J. Phys. Chem. Lett..

[42] Ivanov M., Fedin M.V. (2018). Nanoscale heterogeneities in ionic liquids: Insights from EPR of spin probes. Mendeleev Commun..

[43] Bakulina O.D., Ivanov M.Y., Prikhod’Ko S.A., Pylaeva S., Zaytseva I.V., Surovtsev N.V., Adonin N.Y., Fedin M.V. (2020). Nanocage formation and structural anomalies in imidazolium ionic liquid glasses governed by alkyl chains of cations. Nanoscale.

[44] Ivanov M.Y., Poryvaev A.S., Polyukhov D.M., Prikhod’Ko S.A., Adonin N.Y., Fedin M.V. (2020). Nanoconfinement effects on structural anomalies in imidazolium ionic liquids. Nanoscale.

[45] (2002). PREPARATION OF 1-BUTYL-3-METHYL IMIDAZOLIUM-BASED ROOM TEMPERATURE IONIC LIQUIDS. Org. Synth..

[46] Rajca A., Kathirvelu V., Roy S.K., Pink M., Rajca S., Sarkar S., Eaton S.S., Eaton G.R. (2010). A Spirocyclohexyl Nitroxide Amino Acid Spin Label for Pulsed EPR Spectroscopy Distance Measurements. Chem.—A Eur. J..

[47] Kirilyuk I.A., Polienko Y.F., Krumkacheva O.A., Strizhakov R.K., Gatilov Y.V., Grigor’Ev I.A., Bagryanskaya E.G. (2012). Synthesis of 2,5-Bis(spirocyclohexane)-Substituted Nitroxides of Pyrroline and Pyrrolidine Series, Including Thiol-Specific Spin Label: An Analogue of MTSSL with Long Relaxation Time. J. Org. Chem..

[48] Stoll S., Schweiger A. (2006). EasySpin, a comprehensive software package for spectral simulation and analysis in EPR. J. Magn. Reson..

[49] Kathirvelu V., Smith C., Parks C., Mannan A., Miura Y., Takeshita K., Eaton S.S., Eaton G.R. (2008). Relaxation rates for spirocyclohexyl nitroxyl radicals are suitable for interspin distance measurements at temperatures up to about 125 K. Chem. Commun..

[50] Milov A.D., Samoilova R.I., Shubin A.A., Grishin Y.A., Dzuba S.A. (2008). ESEEM Measurements of Local Water Concentration in D2O-Containing Spin-Labeled Systems. Appl. Magn. Reson..

[51] Syryamina V., Maryasov A., Bowman M., Dzuba S. (2015). Electron spin echo envelope modulation of molecular motions of deuterium nuclei. J. Magn. Reson..

[52] Kirilina E.P., Grigoriev I.A., Dzuba S.A. (2004). Orientational motion of nitroxides in molecular glasses: Dependence on the chemical structure, on the molecular size of the probe, and on the type of the matrix. J. Chem. Phys..

[53] Paschenko S.V., Toropov Y.V., Dzuba S.A., Tsvetkov Y.D., Vorobiev A.K. (1999). Temperature dependence of amplitudes of libration motion of guest spin-probe molecules in organic glasses. J. Chem. Phys..

[54] Dzuba S. (2000). Libration motion of guest spin probe molecules in organic glasses: CW EPR and electron spin echo study. Spectrochim. Acta Part A Mol. Biomol. Spectrosc..

[55] Kuzhelev A.A., Strizhakov R.K., Krumkacheva O.A., Polienko Y.F., Morozov D.A., Shevelev G.Y., Pyshnyi D.V., Kirilyuk I.A., Fedin M.V., Bagryanskaya E.G. (2016). Room-Temperature Electron Spin Relaxation of Nitroxides Immobilized in Trehalose: Effect of Substituents Adjacent to NO-Group. J. Magn. Reson..

[56] Isaev N.P., Dzuba S.A. (2008). Fast Stochastic Librations and Slow Rotations of Spin Labeled Stearic Acids in a Model Phospholipid Bilayer at Cryogenic Temperatures. J. Phys. Chem. B.

