# Supplementary material for: Peek Inside the Water Mixtures of Ionic Liquids at Molecular Level: Microscopic Properties Probed by EPR Spectroscopy
Source: Int J Mol Sci. 2021 Nov 2;22(21):11900. doi: 10.3390/ijms222111900 (PMC8584414; doi:10.3390/ijms222111900)
Supplement: Supplementary file 1 [file ijms-22-11900-s001.zip › ijms-1438564-supplementary.pdf]

International Journal of *Molecular Sciences*

MDPI

1. Synthesis of ionic liquid [Bmim]BF_4

The ionic liquid [Bmim]Cl was obtained by analogy with the literature method from corresponding nitrogen-containing compound 1-methyl imidazole and butyl chloride [45].

A 100 mL round-bottomed flask fitted with magnetic stirrer bar and reflux condenser was charged by 26.5 mmol [Bmim]Cl, 53 mmol of corresponding salt $NaBF_4$ and 80 mL of acetone. The mixture was stirred for 3 h at 50 °C. The solid was filtered and washed by 10 mL of acetone. The second portion of corresponding salt (20 mmol) was added to the filtrate and the resulting mixture was stirred for 3 h at 50 °C. The solid was filtered and washed by 10 mL of acetone. The filtrate was evaporated in vacuum. The residue was solved in 30 mL of dry dichloromethane (to remove NaCl and excess of starting salt) and filtered. The filtrate was evaporated and product was dried in high vacuum (10^{-3} bar) at 80 °C for 6 h. Yields of products are 75–85 %. The reaction of sample of ionic liquid with $AgNO_3$ was negative, that demonstrates the absence of Cl– ions in the ionic liquid media. The NMR spectra were recorded on a Bruker AVANCE 300 spectrometer (^1H at 300.13 MHz, ^{19}F at 282.40 MHz). The chemical shifts are referenced to TMS (^1H), CCl3F (^{19}F, with C_6F_6 as secondary reference (−162.9 ppm))

1. -Butyl-3-methylimidazolium tetrafluoroborate ([Bmim]BF_4)

^1H NMR ($CDCl_3$): δ 8.83 (s, 1H, H-2); 7.31 (s, 1H, H-4); 7.26 (s, 1H, H-5); 4.16 (t, 2H, $^3J_{HH}$ 7.4 Hz, α-CH_2); 3.94 (s, 3H, NCH_3); 1.84 (tt, ^2H, $^3J_{HH}$ 7.4 Hz, $^3J_{HH}$ 7.4 Hz, β-CH_2); 1.35 (qt, 2H, $^3J_{HH}$ 7.4 Hz, $^3J_{HH}$ 7.5 Hz, γ-CH_2); 0.84 (t, 3H, $^3J_{HH}$ 7.4 Hz, δ-CH_3). ^{19}F NMR ($CDCl_3$): δ -152.67 (s, 4F, BF_4).

2. Synthesis of the spin probes K1 and K2

14-Cyano-7-azadispiro[5 .1.5.2]pentadeca-14-ene-7-oxyl (K2) was prepared according to the literature protocol [47]; 2,4-dioxo-1,3,13-triazatrispiro[4.1.5.1.5.1]icosan-13-oxyl (K1) was synthesized as described below using slightly modified literature method [46].

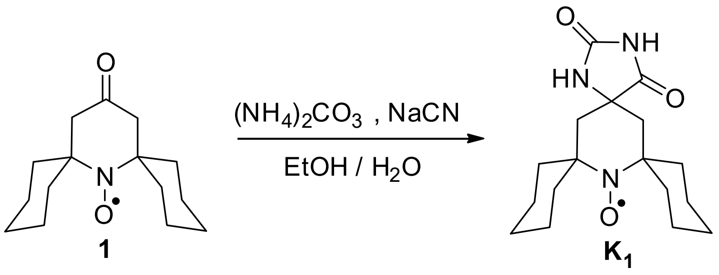

Solution of $(NH_4)_2CO_3$ (300 mg, 3.1 mmol) in distilled water (2 mL) was added to a solution of nitroxide **1** (150 mg, 0.6 mmol) in ethanol (5 mL), and solution of NaCN (100 mg, 2.0 mmol) in distilled water (1 mL) was added dropwise to the mixture upon stirring. The reaction mixture was heated to ~ 70 °C and stirred for 30 h. Then ethanol was distilled off, and the resulting mixture was extracted with ethyl acetate. The organic extract was washed with brine, a solvent was distilled off under reduced pressure, and the residue was subjected to column chromatography on silica gel, eluent hexane–ethyl acetate (1:1), to give K_1: yield 175 mg (91%), orange crystals. Crystallization of K_1 from chloroform leads to the formation of pink crystals of a crystalline solvate with chloroform, mp 241-242 °C.

^1H NMR spectrum of nitroxide K1 was recorded after reduction to corresponding amine with Zn/TFA. To the solution of the nitroxide (15 mg) in CD_3OD (250 μL) Zn powder (100 mg) was added and the mixture was heated to gentle reflux under stirring. Then

TFA (100 μL) was added dropwise and mixture was heated under stirring maintaining the reflux for 15 min, filtered into NMR tube and diluted to required volume with $CDCl_3$. IR spectrum was acquired on FT-IR spectrometer in KBr and is reported in wave numbers (cm^{-1}).

IR ν_{max} 3494, 3340, 3268, 3168, 3029, 2956, 2929, 2861, 2775, 1772, 1716, 1452, 1425, 1403, 1353, 1326, 1255, 1241, 1178, 1164, 1147, 1112, 1033, 914, 759, 750, 690, 651. 1H NMR (300 MHz; ^1H NMR (300 MHz; $CDCl_3/CD_3OD/CF_3COOH$, δ): 1.15-1.32 (m, 2H), 1.32-1.52 (m, 4H), 1.58-1.84 (m, 10H), 1.91-2.03 (m, 2H), 2.08 and.2.28 (AB, *J* = 14.7 Hz, each 2H), 2.20-2.37 (m, 2H). Anal. Calcd for $C_{17}H_{26}N_3O_3{\times}CHCl_3$: C, 49.16; H, 6.19; N, 9.55. Found: C, 49.04; H, 5.99; N, 9.41.

3. Auxiliary ESEEM data

Figure S1 shows the three-pulse ESEEM spectra obtained for K1 and K2 radicals in $[Bmim]BF_4$ and, for reference, the same spectra obtained for another IL $[Bmim]PF_6$, as well as for toluene solution. All spectra were recorded using the same settings ($\pi/2 - \tau - \pi/2 - T - \pi/2$ – echo, 10 ns pulses, τ = 180 ns, T = 600 ns) at 20 K. Such set of ESEEM spectra allows unambiguous assignment of all peaks. Indeed, the strongest peak at ~1.6 MHz is present for K1/K2 in $[Bmim]BF_4$, but it is absent in $[Bmim]PF_6$ and toluene; therefore it is safely assigned to ^{10}B, and its gyromagnetic ratio fits perfectly to yield this value at X-band. Minor ^{31}P, ^{11}B and ^{19}F peaks are also present at suitable frequencies; however, these are not used for any conclusions in this work. The strong proton peaks at ~14.9 MHz are always present and mainly owe to the own protons of radicals.

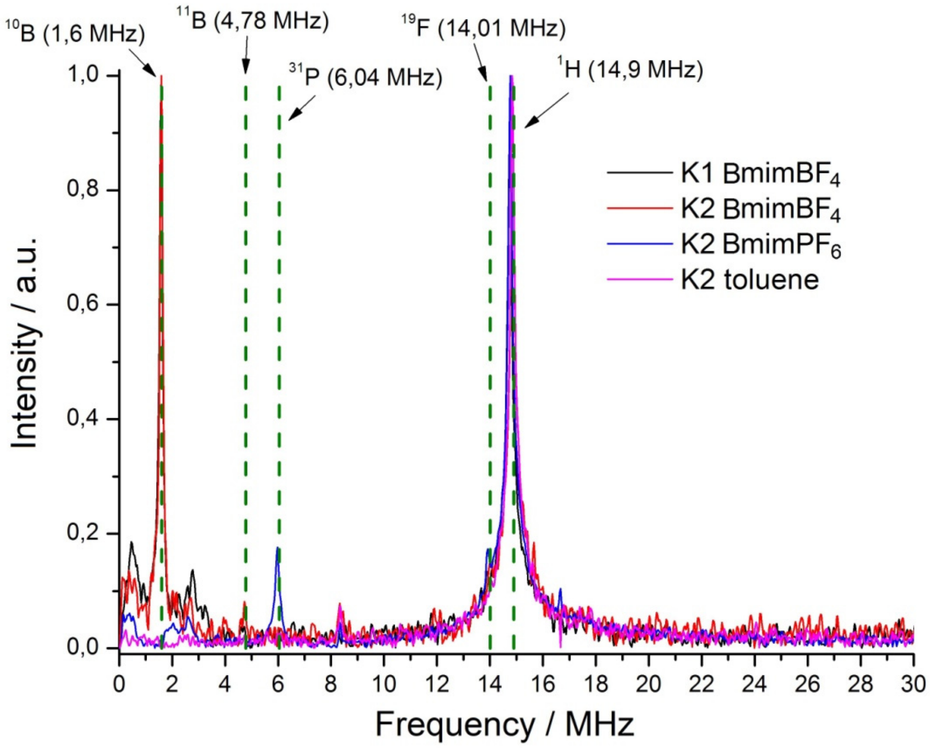

Figure S1. Three-pulse ESEEM spectra measured for K1/K2 in different solvents (indicated in the legend). All data are normalized to the ^1H peak. No water is present in any sample.

4. Continuous wave EPR spectra simulation

The computer simulations were performed using EasySpin [48]. Spectral parameters used in simulations are listed in the Tables S1–S4. In simulations the three following parameters were varied: parallel component of the hyperfine interaction tensor A_{ZZ}, correlation time τ_c (only for 300 K) and spectral linewidth (Gaussian). The values of A_{XX}=A_{YY} components for K1 and K2 were taken as 16.25 MHz and 14.01 MHz, respectively, and g-tensors were taken as [2.0084 2.0064 2.0024] and [2.0074 2.0054 2.0017], respectively, for similar radicals studied in the literature [55].

Table S1. List of parameters used in simulation of CW EPR spectra of K1 in [Bmim]BF_4/D_2O mixture at 300 K.

D_2O wt%	lw / MHz	log(τ_c)	A_{ZZ} / MHz
0.2	0.47	−8.40	94.3
5	0.40	−8.90	94.4
10	0.40	−9.21	95.3
15	0.40	−9.37	96.1
20	0.40	−9.48	96.6
40	0.38	−9.69	98.0
100	0.40	−10.45	104.0

Table S2. List of parameters used in simulation of CW EPR spectra of K1 in [Bmim]BF_4/D_2O mixture at 130 K.

D_2O wt%.	lw / MHz	A_{ZZ} / MHz
0.2	0.89	96.7
5	0.90	97.8
40	0.87	97.2

Table S3. List of parameters used in simulation of CW EPR spectra of K2 in [Bmim]BF_4/D_2O mixture at 300 K.

D_2O wt%	lw / MHz	log(τ_c)	A_{ZZ} / MHz
0.2	0.47	−9.02	93.4
5	0.46	−9.46	94.1
10	0.46	−9.67	95.0
15	0.46	−9.83	95.6
20	0.46	−9.87	96.0
40	0.44	−10.07	97.0
100	0.47	−10.40	102.7

Table S4. List of parameters used in simulation of CW EPR spectra of K2 in [Bmim]BF_4/D_2O mixture at 130 K.

D_2O wt%	lw / MHz	A_{ZZ} / MHz
0.2	0.83	94.9
5	0.83	94.5
40	0.82	94.5

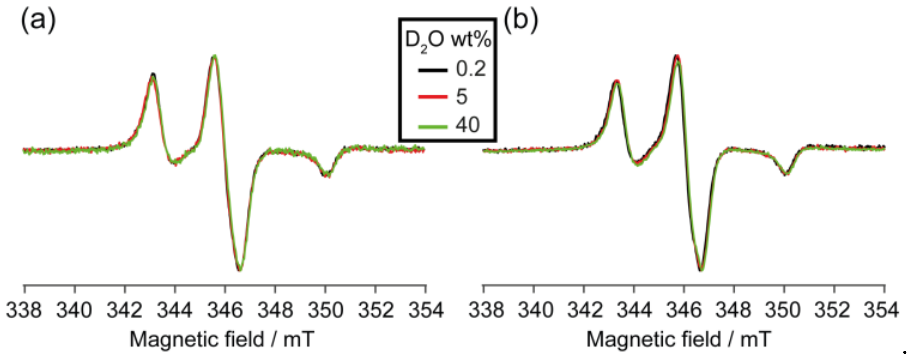

Figure S2. CW EPR spectra of K1 (**a**) and K2 (**b**) nitroxide radicals in [Bmim]BF_4/D_2O mixture at 130 K.

The data of CW EPR at 130 K (frozen glassy solution) shown in Figure S2 and parameters listed in Tables S2 and S4 show that A_{zz} value is not very sensitive to the presence of water for both K1 and K2. Therefore, the data obtained by ESEEM is more informative for the tasks of this work.

6. EPR of stochastic molecular librations in a nutshell

EPR is a very useful tool to investigate the mobility of paramagnetic molecules in various media. In particular, stochastic molecular librations of molecules commonly occur in molecular glasses. They represent the small-angle wobbling of molecules driven by thermal energy. When spin probe, most often the nitroxide radical, is placed in glass, it exhibits similar librations to those occurring in the surrounding matrix. In other words, being tightly embedded in the glass, the spin probe follows its molecular mobility/wobbling. This makes spin probes in the glasses true reporters of the state of the surrounding matrix.

Electron paramagnetic resonance (EPR) is generally sensitive to the mobility of the nitroxide probe. Most commonly, the shape of CW EPR spectrum allows one to determine the rotational correlation times of the nitroxide. However, when the mobility is restricted to small-angle wobbling (e.g., $< 1^{\circ}$), CW EPR becomes poorly sensitive. At the same time, pulse EPR based relaxation methods come into play, because the electron spin relaxation is decently influenced even by such small motions as librations.

A series of works by Dzuba et.al. developed a potent approach to characterize the librations of nitroxide probes in molecular glasses [54,56]. The product $<\alpha^2>\tau_a$ can be experimentally obtained, where $<\alpha^2>$ is the mean squared angular amplitude of motion and τ_a is the corresponding correlation time. This approach is based on the measurements of transverse relaxation times (T_2) of nitroxide in two definite spectral positions. The theoretical consideration of spin relaxation induced by the fast (sub-microsecond) stochastic molecular librations predicts the exponential decay of the two-pulse electron spin echo (ESE) signal upon incrementing the time delay between two pulses. The decay rate is determined by spectral anisotropy at the position of the nitroxide spectrum (positions I and II in Fig.S3a). The nitroxide spectrum is split into three lines due to the hyperfine interaction (HFI) between the spin of unpaired electron and the spin of nitrogen nucleus (I=1). The central component (I) is influenced only by the anisotropy of the g-tensor, with the anisotropy of HFI being negligible; therefore, it possesses the smallest anisotropy and the narrowest linewidth. For the broadest high-field component (II), both the anisotropies influence the spectral shape in an additive way, so it is the most anisotropic and the broadest one. Therefore, the decay rate for the field positions (I) and (II) is essentially different. However, there are other additive relaxation mechanisms contributing to the resulting T_2

values. Therefore, in order to elucidate pure libration-induced relaxation, one should subtract the relaxation rates (inverse relaxation times T_2) in the two spectral positions (I) and (II).

Redfield relaxation theory shows that for fast (sub-microsecond) and small-angle librations the difference in phase memory relaxation rates ($\Delta W \equiv 1/T_2^{(II)}$- $1/T_2^{(I)}$) can be estimated for nitroxide spin probe as $\Delta W = C<\alpha^2>\tau_a$, where $<\alpha^2>$ and τ_a were defined above, and C is the numerical coefficient, whose value was semi-empirically determined to be 9 × 10^{16} s^{-2} [56]. In Dzuba's studies the value $10^{11}<\alpha^2>\tau_c$ is used to analyze the librations of spin probes at different temperatures [54,56]. The multiplier 10^{11} is chosen only for the simplicity because the final value of $10^{11} < \alpha^2 > \tau_c$ is close to 1 what makes it comfortable to analyze data in the plot. However, technically not every point at the temperature dependence of $10^{11} < \alpha^2 > \tau_c$ plot can be assigned to librations. At higher temperatures the radical undergoes high amplitude motion that can be determined as the diffusive rotational motion, where libration theory cannot be applied. Nevertheless, it is convenient to use one plot and one parameter for the entire temperature dependence keeping in mind the applicability range of the libration model. For this reason the L parameter has been introduced. Formally, it is defined as $L \equiv \Delta W \times 10^{11}/C$, which coincides with $10^{11} < \alpha^2 > \tau_c$ in appropriate temperature regime. According to its physical meaning, L has units of rad^{2}s. In our experiments we estimate the relative error of L value as 3 %.

However, not the absolute value of L at certain temperature, but the shape of the $L(T)$ dependence is most informative and has to be analyzed. Theory of atomic displacements predicts that $L(T)$ should linearly grow with temperature. Figure S3b sketches possible behaviors of $L(T)$ dependence. The onset of librations indicates the temperature where librations start to influence electron spin relaxation strong enough to be detected in ESE-based T_2 measurements. The slope of the $L(T)$ curve characterizes the intensity of librations. When glass softens and transforms into a liquid, the amplitude of the nitroxide motions drastically grows, leading to a steep rise of $L(T)$ until T_2 becomes too short to be measured.

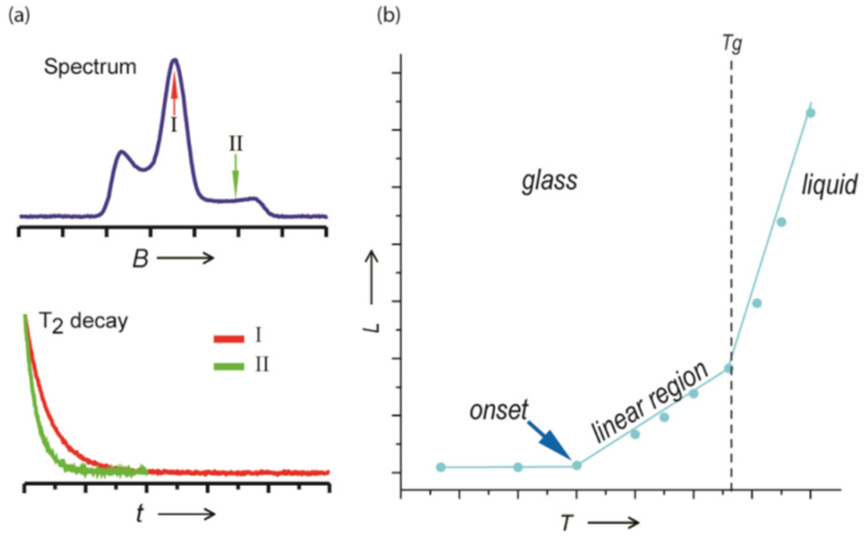

Figure S3. Sketch of the pulse EPR analysis scheme. (**a**) Two-pulse electron spin echo decay is measured by incrementing τ delay in "$\pi/2 - \tau - \pi$ – echo" sequence at two spectra positions I and II. Corresponding T_2 times are obtained by monoexponential analysis. (**b**) Typical $L(T)$ dependence showing low-temperature region of no librations, then their onset, linear region of effective librations, and finally transition into a liquid state with steep increase of molecular motion.

7. FE-SEM images analyses

We have analyzed the FE-SEM images of [Bmim]BF_4/H_2O mixtures studied in Ref [28]. For this sake, we employed MatLab© image processing functions. All water rich domains were pointed, we estimated the visible water concentration and compared it with that mentioned by authors.

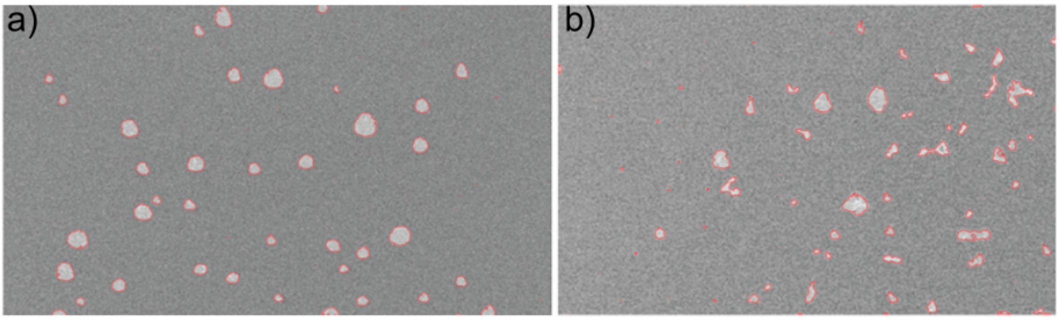

Figure S4. FE-SEM image of [Bmim]BF_4/H_2O mixture with different water concentrations: (**a**) "traces of water", (**b**) 5 wt% of water [28]. The borders of water-rich domains are depicted with red lines; their area has been calculated in analyses.

According to Ref. [28], Figure S4a corresponds to "traces of water", Figure S4b to 5% of water. Our estimations from image analyses gave values of 3%–3.1% water for each case.

References

45. Dupont, J.; Consorti, C. S.; Suarez, P. A. Z.; de Souza, R. F. "Preparation of 1-Butyl-3-Methyl Imidazolium-Based Room Temperature Ionic Liquids," in *Organic Syntheses*, Hoboken, NJ, USA: John Wiley & Sons, Inc., **2003**, pp. 236–236.
47. Kirilyuk, I.A.; Polienko, Y.F.; Krumkacheva, O.A.; Strizhakov, R.K.; Gatilov, Y. V.; Grigor'ev, I.A.; Bagryanskaya, E.G. Synthesis of 2,5-Bis(spirocyclohexane)-Substituted Nitroxides of Pyrroline and Pyrrolidine Series, Including Thiol-Specific Spin Label: An Analogue of MTSSL with Long Relaxation Time. *J. Org. Chem*. **2012**, *77*, 8016–8027.
46. Rajca, A.; Kathirvelu, V.; Roy, S.K.; Pink, M.; Rajca, S.; Sarkar, S.; Eaton, S.S.; Eaton, G.R. A Spirocyclohexyl Nitroxide Amino Acid Spin Label for Pulsed EPR Spectroscopy Distance Measurements. *Chem. Eur. J.* **2010**, *16*, 5778–5782.
48. Stoll, S.; Schweiger, A. EasySpin, a Comprehensive Software Package for Spectral Simulation and Analysis in EPR. *J. Magn. Reson.* **2006**, *178* (1), 42–55.
55. Kuzhelev, A. A.; Strizhakov, R. K.; Krumkacheva, O. A.; Polienko, Y. F.; Morozov, D. A.; Shevelev, G. Y.; Pyshnyi, D. V.; Kirilyuk, I. A.; Fedin, M. V.; Bagryanskaya, E. G. Room-Temperature Electron Spin Relaxation of Nitroxides Immobilized in Trehalose: Effect of Substituents Adjacent to NO-Group. *J. Magn. Reson.* **2016**, *266*, 1–7.
54. Dzuba, S. A. Libration Motion of Guest Spin Probe Molecules in Organic Glasses: CW EPR and Electron Spin Echo Study. *Spectrochim. Acta - Part A Mol. Biomol. Spectrosc.* **2000**, *56* (2), 227–234.
56. Isaev, N. P.; Dzuba, S. A. Fast Stochastic Librations and Slow Rotations of Spin Labeled Stearic Acids in a Model Phospholipid Bilayer at Cryogenic Temperatures. *J. Phys. Chem. B* **2008**, *112* (42), 13285–13291.
28. Kashin, A. S.; Galkin, K. I.; Khokhlova, E. A.; Ananikov, V. P. Direct Observation of Self-Organized Water-Containing Structures in the Liquid Phase and Their Influence on 5-(Hydroxymethyl)Furfural Formation in Ionic Liquids. *Angew. Chem. Int. Ed.* **2016**, *55* (6), 2161–2166.
